# A Comparative Analysis of the Protein Cargo of Extracellular Vesicles from Helminth Parasites

**DOI:** 10.3390/life13122286

**Published:** 2023-11-30

**Authors:** María Eugenia Ancarola, Lucas L. Maldonado, Lucía C. A. García, Gisela R. Franchini, Gustavo Mourglia-Ettlin, Laura Kamenetzky, Marcela A. Cucher

**Affiliations:** 1 Department of Microbiology, School of Medicine, University of Buenos Aires, Buenos Aires C1121, Argentina; meancarola@gmail.com (M.E.A.); lucas.l.maldonado@gmail.com (L.L.M.);; 2 Institute of Research on Microbiology and Medical Parasitology (IMPaM, UBA-CONICET), University of Buenos Aires, Buenos Aires C1121, Argentina; 3 Instituto de Tecnología (INTEC), Universidad Argentina de la Empresa (UADE), Buenos Aires C1073, Argentina; 4 Instituto de Investigaciones Bioquímicas de La Plata (INIBIOLP), Facultad de Ciencias Médicas, Universidad Nacional de La Plata (UNLP)-Consejo Nacional de Investigaciones Científicas Y Técnicas (CONICET), La Plata B1900, Argentina; gfranchini@gmail.com; 5 Departamento de Ciencias Biológicas, Facultad de Ciencias Exactas, Universidad Nacional de La Plata (UNLP), La Plata B1900, Argentina; 6 Área Inmunología, Departamento de Biociencias, Facultad de Química, Universidad de la República, Montevideo 11800, Uruguay; gmourglia@higiene.edu.uy; 7 Instituto de Biociencias, Biotecnología y Biología Traslacional, Departamento de Fisiología y Biología Molecular y Celular, Facultad de Ciencias Exactas y Naturales, Universidad de Buenos Aires, Buenos Aires C1428, Argentina; lauka@fbmc.fcen.uba.ar

**Keywords:** helminth, parasite, extracellular vesicle, protein, marker

## Abstract

Helminth parasites cause debilitating—sometimes fatal—diseases in humans and animals. Despite their impact on global health, mechanisms underlying host–parasite interactions are still poorly understood. One such mechanism involves the exchange of extracellular vesicles (EVs), which are membrane-enclosed subcellular nanoparticles. To date, EV secretion has been studied in helminth parasites, including EV protein content. However, information is highly heterogeneous, since it was generated in multiple species, using varied protocols for EV isolation and data analysis. Here, we compared the protein cargo of helminth EVs to identify common markers for each taxon. For this, we integrated published proteomic data and performed a comparative analysis through an orthology approach. Overall, only three proteins were common in the EVs of the seven analyzed species. Additionally, varied repertoires of proteins with moonlighting activity, vaccine antigens, canonical and non-canonical proteins related to EV biogenesis, taxon-specific proteins of unknown function and RNA-binding proteins were observed in platyhelminth and nematode EVs. Despite the lack of consensus on EV isolation protocols and protein annotation, several proteins were shown to be consistently detected in EV preparations from organisms at different taxa levels, providing a starting point for a selective biochemical characterization.

## 1. Introduction

Pathogens and hosts communicate using a diverse range of signals that can involve secreted factors. In chronic infections, such as those produced by helminth parasites, this exchange of molecules may occur for years and allows pathogens to modulate the surrounding environment for their own benefit. The helminth parasites that affect human and livestock health are mainly classified within the phyla Platyhelminthes (flatworms) and Nematoda (roundworms). Helminths is a general term used to group these two taxa, which comprise both free-living and parasitic species; however, these phyla are composed of highly divergent species that do not share a common evolutionary origin. Nematodes are classified into five major clades (I to V), all of which contain parasites; while main parasitic Platyhelminthes include the classes Trematoda and Cestoda (also known as flukes and tapeworms, respectively). Overall, helminths are the causative agents of multiple debilitating, sometimes fatal, diseases in humans, as well as domestic and wild fauna. Globally, it is estimated that ~16% of the human population is infected with helminth parasites [1]. Some of these pathogens are mostly associated with poverty and affect the most vulnerable and marginalized populations in the world. Many of the diseases they cause can be mostly prevented via access to basic water supply and sanitation facilities that allow for the undertaking of proper hygiene practices [2]. Despite their impact on global health, worm biology research is underfunded and consequently, the study of helminthiases has historically lagged behind in comparison to other infectious diseases. This is reflected in the fact that there is still a large gap in our understanding of the molecular and cellular mechanisms underlying host–parasite interactions, which are fundamental to the control and eradication of these diseases.

More than a decade ago, the fact that helminth parasites secrete extracellular vesicles (EVs) that can be internalized by host cells was described for the first time [3]. EVs are non-replicative subcellular nanoparticles that are enclosed in a lipid bilayer and can be secreted by any type of cell. EV secretion is a conserved mechanism throughout evolution and has been described in all kingdoms of life [4]. There are multiple types of EVs that differ in their biogenesis, release, size, content and density, and can be roughly classified into two groups, namely exosomes and microvesicles [5]. Exosomes are intraluminal vesicles (ILVs) generated through the endosomal pathway by the inward budding of the endosomal membrane. This process gives rise to multivesicular bodies (MVB), which, upon fusion with the plasma membrane, release the exosomes into the extracellular environment [6]. On the other hand, microvesicles bud from the plasma membrane. The different populations of EVs represent subsets of the contents of the cell that originated them; hence, the protein, nucleic acid and lipid profiles are expected to differ among EV types, with a consequent effect on EV functionality. 

To date, EV secretion has been studied in the three main groups of parasitic helminths (cestodes, trematodes and nematodes), including the characterization of EV protein content. The amount of data obtained has shed light on aspects such as the identification of EV-biogenesis pathways [7], surface proteins involved in the interaction with the host [8,9,10,11], sites of EV production [8,12,13], drug targets [13], and diagnosis/vaccine candidates [14,15,16,17,18]. However, this wealth of information is highly heterogeneous, since it was generated in multiple parasite species and/or life cycle stages, using varied protocols for EV isolation and data analysis. On the other hand, as is the case for all of the organisms studied to date, most proteins are inferred via in silico annotation and still lack a proper biochemical and functional characterization [19]. In addition, protein annotation varies depending on the pipeline used by each genomic consortium, since there are no common criteria to follow and, in many cases, the input data employed to train the algorithms introduce bias into the results obtained [20]. Consequently, data integration is not a simple task and requires employing a unifying approach to perform comparative studies, as previously done with functionally annotated proteins [7,21].

Genomics and proteomics have provided a high quantity of information about parasite species. However, high-throughput experiments, while providing large amounts of data, often provide information that is not specific enough to be useful for functional classification [22]. Furthermore, parasites express a variable number of proteins for which functionality cannot be assigned due to lack of sequence or domain conservation with other, better studied proteins. These proteins are usually annotated as “hypothetical”, “expressed” or “predicted”, and are mostly disregarded in further studies. In helminth parasites, around half of the proteomes correspond to proteins of unknown function [23]. In the case of pathogens, these proteins may play key roles for survival, establishment and/or communication with hosts and, hence, deserve proper characterization. Hypothetical proteins found in EVs from pathogens may as well represent specific markers with no host cross-reactivity. Such protein markers may be useful for isolating EV subpopulations or determining parasite EV presence in a complex sample, i.e., serum from an infected host. To date, knowledge has been mostly obtained by studying human and mouse EVs; hence, protein markers common to mammalian EV may not be conserved in pathogens. This seems to be the case in protozoans, where tetraspanins are not present in EVs from several species (reviewed in [24]). In this context, annotation by orthology allows for the transfer of biological function through the inference of the common ancestry of proteins that have not been experimentally characterized and/or do not contain conserved functional domains. In this sense, whole proteomes are compared, and orthologous proteins are sorted into the same ortholog group (OG). Furthermore, this allows for the identification of parasite genes, which are highly divergent from those of the host.

The aim of this work was to compare the protein cargo of EVs from helminth parasites and to identify common protein markers for each group (Cestoda, Trematoda and Nematoda), as well as specific markers for each species/genus, which is potentially useful for determining EV presence in complex multi-species samples (e.g., liquid biopsies). For this, we integrated published proteomic data from EVs secreted by parasitic platyhelminths and nematodes and performed a comprehensive comparative analysis through an orthology approach, which allowed us to study the complete proteomes of parasites, i.e., proteins with and without functional annotation.

## 2. Materials and Methods

### 2.1. Orthology Analysis

The OGs were built using OrthoMCL v.14-137, as previously described [25]. Briefly, MySQL 14.14 Distrib 5.7.36 was used for database management, protein sequences were retrieved from UniProt Swissprot (https://ftp.uniprot.org/pub/databases/uniprot/, accessed on 20 March 2018) and the proteomes of parasitic helminth species with high quality genomes (BUSCO score ≥ 65) were downloaded from the Wormbase Parasite Database. When more than one genome project was available for a given species, the project with a higher BUSCO score was chosen. Also, we included the proteomes of the free-living nematode *Caenorhabditis elegans* and the free-living platyhelminth *Schmidtea mediterranea*. The proteomes were downloaded from the databases detailed in Supplementary Table S1.

Proteomes were filtered according to protein length > 10 amino acids, and filtered proteins were searched for reciprocal best hits using BLAST [26], e-value < 1 × 10^−15^ and a query coverage > 50%. OGs were constructed using the OrthoMCL algorithm [27], with an inflation value set in 1.5 to define the final orthologs and paralogs. The OGs generated in this work are available at https://github.com/MCucher/Ortholog_groups.

### 2.2. Collection of Published Proteomic Data from the EVs of Helminth Parasites

**Figure 1:**
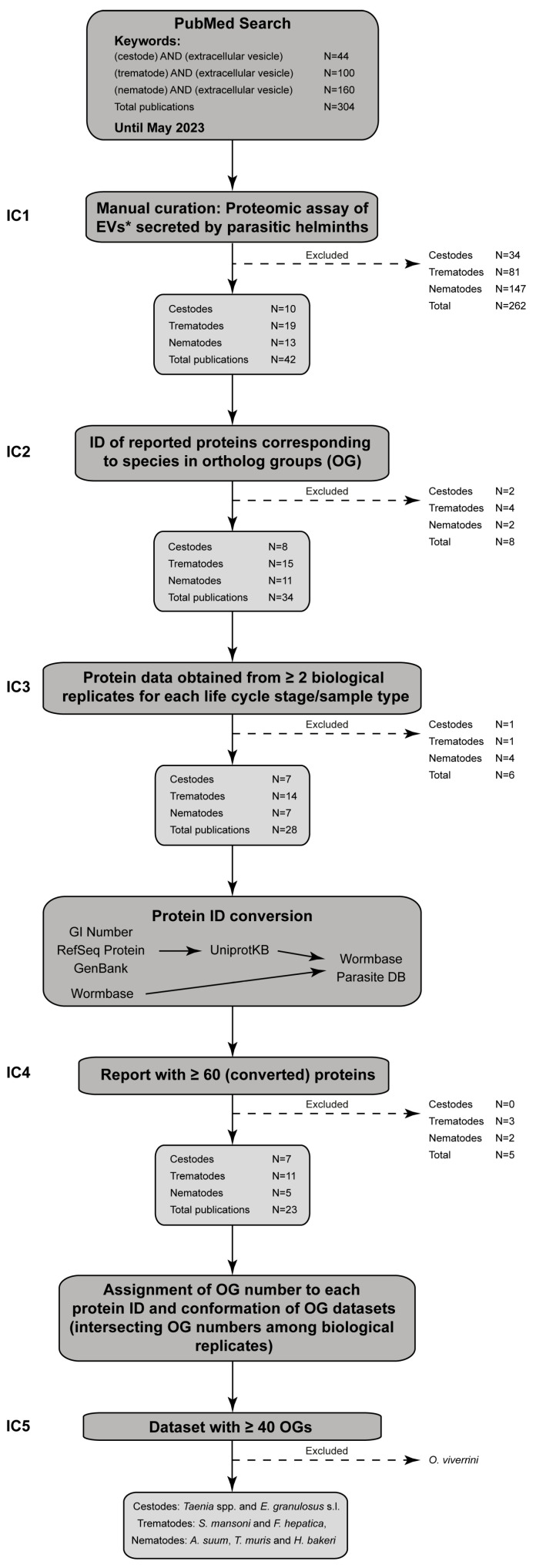
Flow diagram of the data collection, selection and processing of helminth EV proteomes. IC: Inclusion criterion. *: Only the results from EVs obtained without any treatment were used. When EVs were obtained by differential centrifugation, only those collected at ≥100,000× *g* were included in the workflow. The numbers correspond to published articles. IC 1: Article describing the proteomic analysis of parasitic helminth EVs and reporting the IDs of all the identified proteins; IC 2: Protein IDs corresponding to helminth species (or genus in the case of *Taenia* spp.) used for the construction of the OGs used in this work; IC 3: Protein data from a type of sample (parasite stage or inner fluid) for a given species/genus including at least two biological replicates; IC 4: Article containing ≥60 Wormbase protein IDs and IC 5: Protein dataset containing ≥40 OGs shared by at least two biological replicates.

The workflow used in this work is depicted in Figure 1. Three bibliography searches were performed in PubMed using the following keywords: (i) cestode AND extracellular vesicle, (ii) trematode AND extracellular vesicle, (iii) nematode AND extracellular vesicle. Articles available up to 3 May 2023 (N = 304) were taken into consideration. After this initial search, the retrieved articles were manually curated by selecting those where a proteomic analysis of parasitic helminth EVs was performed and where the IDs of all the identified proteins were displayed (Inclusion Criterion 1). No distinction based on the methodology used for EV purification was performed; however, when differential centrifugation was used, only protein IDs obtained from 100,000–120,000× *g* centrifugation pellets were retrieved. When EVs from parasites under different treatments were obtained, IDs from control (untreated) parasites were used. The samples used in this work correspond to EVs secreted to the extra-parasite medium (i.e., culture medium) but also to the inner fluid of certain parasites (i.e., hydatid fluid from *E. granulosus* s. l. and body fluid from *A. suum*). In cases where proteins from EV membranes and lumen were studied separately, the protein IDs were collapsed. When authors reported results from multiple biological replicates and/or purification protocols, protein IDs common to all the datasets were retained.

Only protein IDs corresponding to helminth species (or genus in the case of *Taenia* spp.) used for the construction of the OGs were included in this analysis (Inclusion Criterion 2). Finally, protein data from the same type of sample (parasite stage or inner fluid) for a given species/genus were retained if data from at least two biological replicates were reported (Inclusion Criterion 3).

### 2.3. Data Processing

For this analysis, protein IDs from the Wormbase Parasite DB were used. When necessary, the originally reported IDs were transformed to the UniProtKB format, using the Retrieve/ID mapping tool from the UniProt database (https://www.uniprot.org/uploadlists/ (accessed on May–June 2023)). Then, UniProtKB IDs were transformed to the Wormbase Parasite DB format, using the corresponding annotation file from each species. Reports containing ≥60 protein IDs were retained (Inclusion Criterion 4), and the corresponding OG number was assigned to each Wormbase Parasite DB ID. Finally, OGs shared by at least 2 biological replicates from the same species/genus and life cycle stage/sample type were retained. Only datasets containing ≥40 OGs were further analyzed (Inclusion Criterion 5). In this sense, data from 7, 9 and 5 articles corresponding to EVs from cestodes, trematodes and nematodes, respectively, were used (Supplementary Tables S2 and S3).

The protein description of each OG was based on *E. multilocularis* functional annotation. When this species was not present in the OG, the functional annotation from one of the species analyzed in this work was used. 

### 2.4. Proteins of Unknown Function

Proteins with description terms such as Hypothetical protein, Predicted protein, Expressed protein, Tegumental protein or another ambiguous description were considered of unknown function.

Signal peptide prediction was performed with SignalP version 5.0 [28], and transmembrane domain prediction was performed with TMHMM version 2.0 [29]. 

### 2.5. Graphics 

Intersection and dot plot graphics were performed using “UpSet R” package v1.4.0 and ggplot2, respectively, in R Studio v1.4.1103 [30].

## 3. Results and Discussion

After a thorough bibliographic search, 42 reports describing the protein content of EVs from helminth parasites were retained (Figure 1 and Supplementary Table S2). However, EV purification methodologies, protein identification platforms and protein annotation algorithms differed among laboratories, adding variability to the results obtained. Furthermore, numerous works describe the proteomic analysis of only one biological sample. In this sense, to obtain more robust protein datasets, only those proteins identified in at least two independent proteomic assays from the same life cycle stage from a given species/genus were included in the analysis. Finally, to identify common proteins among studies, a comparison was performed through the OG numbers present in each dataset, since orthologous proteins are inferred to proceed from a common ancestor and to have a higher probability of retaining the same biological function. As a result, the following data from representative organisms from each helminth group were collected: (i) Cestoda: *Echinococcus granulosus sensu lato* [31,32,33,34,35] and *Taenia* spp. [36,37], (ii) Trematoda: *Schistosoma mansoni* [14,38,39,40,41] and *Fasciola hepatica* [9,42,43,44] and (iii) Nematoda: *Trichuris muris* [45,46,47], *Ascaris suum* [48] and *Heligmosomoides bakeri* (renamed from *Heligmosomoides polygyrus* [49]) [50] (Figure 1 and Supplementary Table S3). The data used to carry out all the analyses are detailed in Supplementary Table S4.

### 3.1. EVs from Helminth Parasites Share a Core Set of Proteins Secreted in a Taxon-Related Manner

**Figure 2:**
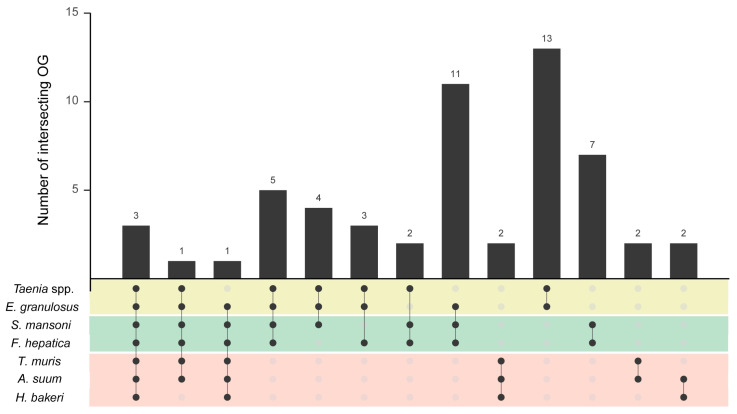
Protein content of extracellular vesicles from helminth parasites. Proteins were classified into ortholog groups (OG), and only OGs shared by taxonomically related groups are displayed. Black and grey dots indicate presence or absence, respectively, in the genus/species described in the corresponding row. The connecting line indicates intersection between parasites with black dots, and the number on a bar indicates the number of intersecting OGs. Colored panels indicate the taxon: yellow for Cestoda, green for Trematoda and red for Nematoda.

As a first general approach, the total protein content displayed by the EVs from each parasite (i.e., all the proteins detected in the EVs from all the life cycles stages or sample types for a given helminth) was compared, showing that few proteins are secreted in common in the three groups of parasites: Actin (OG1.5_000110), Heat shock protein 90 (OG1.5_000216) and Fructose-bisphosphate aldolase (OG1.5_001292) (Figure 2 and Supplementary Table S5). Also, two proteins were present in all except one parasite group: Heat shock protein 70 (OG1.5_000150) and Enolase (OG1.5_000805). All these proteins are considered EV markers conserved across evolution in eukaryotes [51]; hence, it is expected that they would be detected in EVs from worms not related phylogenetically, as Nematodes and Platyhelminthes.

The proteins specifically detected in each taxon amounted to 6 in Nematoda (considering its presence in ≥2 species), 25 in Platyhelminthes (considering its presence in ≥3 species), 13 in Cestoda and 7 in Trematoda (Figure 2 and Table 1). Interestingly, even though the *Taenia* dataset contains the lowest number of proteins (N = 40), the EVs from the parasites of class Cestoda presented the highest number of proteins in common. This result reflects the high phylogenetic conservation of the genera *Echinococcus* and *Taenia*, which belong to the same family (Taeniidae), in contrast to the analyzed species from Trematoda and Nematoda, which belong to different Orders.

Interestingly, taxon-specific proteins with no orthologues in host species (human/mouse) were detected in all parasite groups, including proteins conserved with free- living helminth species, or only present in parasites (Table 1). The secretion of parasite-specific proteins urges a comprehensive characterization to assess the relevance of EV-cargo in the adaptation to a parasitic lifestyle and the interaction with the host or with other parasites. 

### 3.2. EVs from Helminth Parasites Contain Canonical and Non-Canonical Proteins Related to Exosome and Microvesicle Biogenesis

The optimal functioning of the processes involved in EV shedding is required to maintain cellular homeostasis. EVs are not only involved in signaling between cells or organisms but also are used to discard waste products and regulate cellular content levels. In addition, blocking steps of EV biogenesis can alter membrane trafficking and compromise tegument ultrastructure, inducing irreversible damage to parasites when exposed to the host immune response. EVs from a number of parasites were shown to modulate the host immune system; thus, the inhibition of EV secretion through drug action may be a determinant for parasite clearance, as was recently proposed for the filarial nematodes *B. malayi* and *Dirofilaria immitis*, where ivermectin treatment inhibits EV release in susceptible strains [13]. On the other hand, it was suggested that anthelmintic sequestration and secretion in EVs may represent a detoxification mechanism in *F. hepatica* [52], thus indicating another relevant role of EVs in parasite biology.

Furthermore, parasite EVs may be responsible for serious pathogenic effects in some cases, as in *O. viverrini* infection. This disease is associated with bile duct cancer or cholangiocarcinoma, and *O. viverrini* EVs were shown to induce cholangiocytes to acquire a tumorigenic phenotype in vitro [10]. The silencing of tetraspanin genes produced significant alterations in the ultrastructure of parasite tegument [53] and a 2-log reduction in the production of EVs [11]. Hence, interfering with proteins involved in EV biogenesis may be a useful strategy to diminish this pathology.

**Figure 3:**
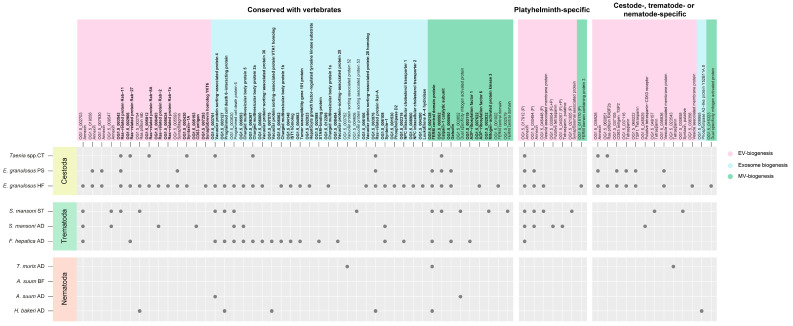
Proteins related to EV-biogenesis detected in EVs from parasitic helminths. Proteins in bold correspond to those described in Bennett et al., 2020 [7]. FL + P: Conserved among free-living and parasitic species; P: Conserved among parasitic species only. Grey dots indicate presence. CT: Cysticercus, PS: Protoscolex, HF: fertile Hydatid Fluid, ST: Schistosomulae, AD: Adult, BF: Body Fluid.

A detailed study on the conservation of the pathways involved in the biogenesis of exosomes and microvesicles in helminth parasites was previously reported [7]. Of the total number of proteins described as involved in EV-biogenesis in model organisms [5,7], 68.8% (75/109) presented orthologues in a diverse number of helminth parasites (Supplementary Table S6). From these, 28, 22 and 5 proteins were detected in the EVs from cestodes, trematodes and nematodes, respectively (Figure 3, OGs in bold type). In addition, proteins containing functional domains corresponding to those involved in EV-biogenesis were also detected. However, the sequence divergence presented between parasite and model organism proteins may have prevented them from being clustered in the same OGs. These proteins accounted for 20, 17 and 5 in the EVs from cestodes, trematodes and nematodes, respectively (Figure 3). Interestingly, 20 of these proteins did not present human/mouse orthologues, including 13 that were specific to each parasite group and 7 that were only Platyhelminth-specific (Figure 3). Among the latter, 2 proteins were conserved with planarians (OG1.5_030904 and OG1.5_040267) and 5 were parasite specific (OG1.5_017912, OG1.5_030494, OG1.5_045448, OG1.5_021305 and OG1.5_015170). Regarding the two Nematode-specific proteins, both were conserved with *C. elegans*. In Platyhelminth EVs, annexin (OG1.5_017912) was detected in all the species and samples, while in nematode EVs, no protein was found in common among the three species. Only the FERM central domain protein (OG1.5_002138) was present in both *T. muris* and *H. bakeri* samples. These results suggest a distinctive repertoire of proteins involved in EV generation, with components that may differ between free-living and parasitic Platyhelminthes.

### 3.3. Helminth EVs Carry Taxon-Specific Proteins of Unknown Function

Proteins of unknown function were detected in all the datasets (Supplementary Table S7). This type of proteins are found in all the model and non-model organisms studied to date, and their sequence contains no homology with any described functional domain or protein. Thus, their role in the biology of the species under study remains to be determined. Proteins of unknown function can then be evolutionarily conserved among distant taxa or can be taxon specific. In this sense, pathogen-specific proteins denote unique evolutionary traits, with great potential as EV markers, since they may present low to null cross-reactivity with host proteins. Of particular interest are membrane proteins, which contain surface-exposed domains that can aid in the specific isolation and purification of EVs, which is especially useful in the detection of parasite EVs in complex samples such as a liquid biopsy or an in vitro co-culture medium. On the other hand, parasite-specific proteins secreted towards the extra-parasite milieu may be involved in the adaptation to parasitism, positioning these molecules as attractive novel therapy targets.

**Figure 4:**
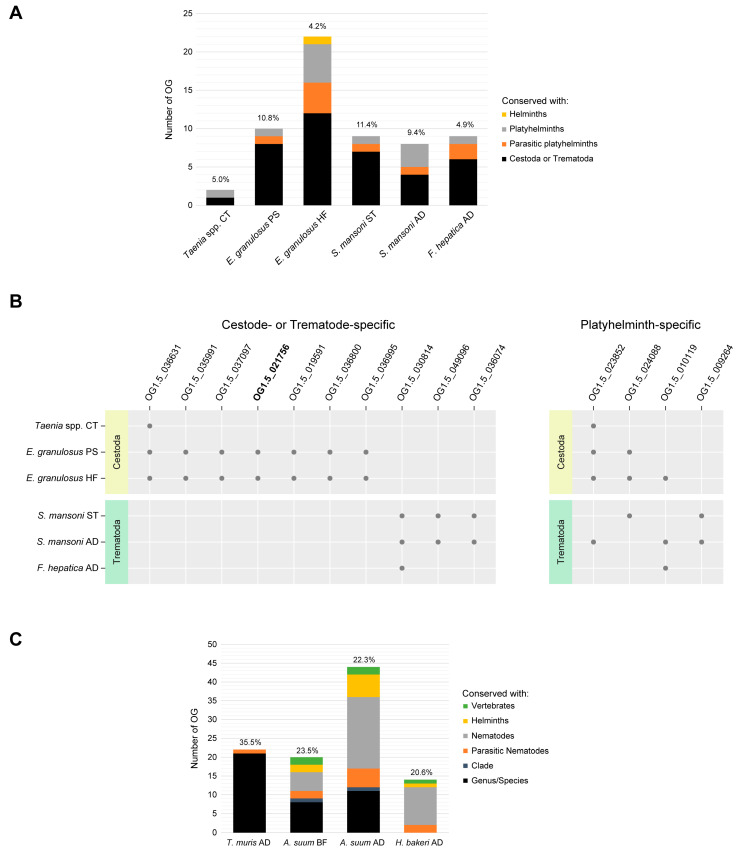
Proteins of unknown function secreted in EVs from parasitic helminths. (**A**) Platyhelminth EVs: Number of ortholog groups (OG) exclusively containing proteins of unknown function. Percentages indicate the proportion with respect to the total number of OGs. (**B**) Proteins of unknown function with no human/mouse orthologues secreted by at least two parasite stages and/or sample types of platyhelminths. OG in bold: protein with transmembrane domains. Grey dots indicate presence. CT: Cysticercus, PS: Protoscolex, HF: fertile Hydatid Fluid, ST: Schistosomulae, AD: Adult. (**C**) Nematode EVs: Number of OGs exclusively containing proteins of unknown function. Percentages indicate the proportion with respect to the total number of OGs. AD: Adult, BF: Body fluid.

In the EVs secreted by Platyhelminth parasites, around 5–10% of the OGs were exclusively composed of proteins of unknown function (Figure 4A). In addition, taxon-specific proteins were detected in common in the EVs from multiple parasites or sample types (Figure 4B). From these, a cestode-specific protein with six transmembrane domains (OG1.5_021756) was detected in *E. granulosus* s. l. EVs, positioning it as a good marker candidate for Cestoda EVs. With respect to Platyhelminth-specific proteins, one (OG1.5_024088) detected in EVs from larval stages in Cestoda and Trematoda is only conserved among parasite species.

Regarding proteins of unknown function from Nematoda, they accounted for 20–35% of total OG numbers (Figure 4C). In most cases, they did not display any human/mouse orthologue. Only the samples from *A. suum* displayed 18 OGs in common, while the remaining were exclusive from each dataset, reinforcing the high divergence among nematode clades (Supplementary Table S7). From the shared proteins, transmembrane domains were detected in 2 conserved within Nematoda (OG1.5_014843 and OG1.5_023612), 1 conserved in parasitic nematodes (OG1.5_017825), 1 conserved within Clade III species (OG1.5_018985) and 3 only belonging to *Ascaris* (OG1.5_034898, OG1.5_043336 and OG1.5_056986). In *T. muris* EVs, all the proteins of unknown function were specific of Trichuris, except one that was conserved among parasitic nematodes (Supplementary Table S7). Of these, 7 contained transmembrane domains (OG1.5_009842, OG1.5_018560, OG1.5_025116, OG1.5_028247, OG1.5_050997, OG1.5_051298 and OG1.5_074123). Finally, in the EVs from *H. bakeri* adults, from the 10 unannotated proteins that presented orthologues in free-living and parasitic nematode species, 1 had transmembrane domains (OG1.5_019313), while of the 2 proteins that were exclusively of parasitic nematodes, only one displayed transmembrane domains (OG1.5_011149).

### 3.4. Helminth Parasites Secrete Moonlighting Proteins as EV-Cargo 

It has been well documented that proteins that display a fundamental role in intracellular metabolic pathways may exert completely different functions in other locations, such as the plasma membrane—or tegument, in the case of multicellular organisms. This activity is denominated as moonlighting to indicate the very different actions that proteins can perform depending on their environment. Moonlighting activity has been reported for different enzymes that belong to the canonical glycolytic pathway, which have been described as present in the plasma membrane of unicellular organisms or in the tegument of several metazoans [54,55,56]. Among the secreted products analyzed in this work, a wide list of cytosolic enzyme examples was found (Supplementary Table S8), but three members of the glycolytic pathway were systematically present in most datasets. These enzymes are fructose-bisphosphate aldolase, enolase and glyceraldehyde-3-phosphate dehydrogenase (GAPDH), and all of them have been well documented as possessing moonlighting activity in different organisms [57].

First, the enzyme aldolase (OG1.5_001292) was consistently found as a part of the EVs from all the helminths analyzed here (Supplementary Table S8). Evidence shows that this protein is located on the tegument in several parasitic helminths, such as *S. mansoni* [58], *F. hepatica* [59,60], *Clonorchis sinensis* [61], *E. granulosus* s. l. [62], *Onchocerca volvulus* [63], *D. immitis* [64] and *Trichinella spiralis* [65]. Interestingly, surface-exposed aldolase from different pathogens has been described with different moonlighting functions. One of the most common reported activities, together with enolase and GAPDH, is the interaction with and activation of plasminogen, a key component of the host coagulation response [66,67]. This activity is thought to help in parasite migration and its permanence in blood-dwelling stages. Additionally, a proteomic analysis of *Trichuris trichiura* has provided evidence of aldolase presenting immune modulatory effects on peripheral blood mononuclear cells from naive individuals [68]. Also, enolase (OG1.5_000805) has been found in EVs from *E. granulosus* s. l., adult trematodes and nematodes (Supplementary Table S8). Enolase catalyzes the conversion of 2-phosphoglycerate to phosphoenolpyruvate in the glycolytic pathway. As aldolase, eukaryotic enolases have been shown to be multifunctional proteins presenting a variety of activities in addition to the ones performed in the glycolytic pathway [69]. Among these activities, it has also been proven to be a plasminogen receptor in *O. volvulus* as a scaffold protein and as a hypoxia-related protein in tissue cultures [66,70,71]. Finally, the enzyme GAPDH (OG1.5_000796) was found in EVs from *E. granulosus* s. l., adult trematodes and adult *T. muris* (Supplementary Table S8). GAPDH is perhaps the glycolytic enzyme with more moonlighting activities described in all types of organisms (e.g., apoptosis, iron transport, membrane fusion, transcriptional regulation, vesicle transport from the endoplasmic reticulum to the Golgi apparatus and cellular response against oxidative stress and hypoxia) [72]. Notably, strong evidence supports GAPDH as a fusogenic protein in intracellular environments [73,74], a feature potentially relevant for the interaction of EVs with host cells. Regarding moonlighting activity in parasitic helminths, it has been associated with plasminogen binding and with complement C3 inactivation in *H. contortus* [75,76].

To fulfill these activities, these enzymes should present a specific topography in the EVs that is membrane attached and exposed to the exterior or as part of the soluble cargo. In either case, their moonlighting activity may vary. Few studies have deepened into this subject; however, in EVs from adult *F. hepatica*, GAPDH was shown to be membrane associated and exposed, while enolase was present within the vesicular lumen [9].

On the other hand, it is worth mentioning the intriguing example of lipid-binding proteins (LBPs). Parasitic helminths produce an unexpectedly wide range of LBPs that are structurally distinct from those of their hosts. Although they have been extensively characterized from the structural point of view, their exact functions are still unknown. Two functions have been proposed for these carrier proteins: (i) lipid transport within organelles or tissues with specialized external functions, including acquisition and distribution of nutrients; and (ii) the modulation of the host local environment. They are major components of helminth secretions; therefore, they are presumably important for establishing host–parasite interactions and persisting during infection, with some being abundant in certain specialized parasite tissues [77]. The co-isolation of extracellular LBPs with EVs may be due to a technical artefact; hence, only cytosolic LBPs will be mentioned here. In the case of *S. mansoni* and *F. hepatica* EVs, two fatty acid binding proteins (FABP) (OG1.5_005776 and OG1.5_035032) were observed, as well as in *H. bakeri* (OG1.5_005776). FABPs are monomeric intracellular proteins that are found to be highly expressed in tissues with active lipid metabolism. Notably, EmFABP4 (OG1.5_036183) was found in EVs isolated from *E. granulosus* s. l. hydatid fluid (Supplementary Table S8). This member of the family presents a striking feature, being a predicted sequence that is much longer (176 amino acids) than what is expected for a canonical FABP (around 130 amino acids). In this case, a typical FABP fold is predicted, but no specific structure is assigned to the C terminus of the protein [78]. Whether this C terminus might be interacting with lipids or other proteins found in EVs remains to be elucidated. 

### 3.5. Parasitic Platyhelminths and Nematodes Secrete EVs with Different Repertoires of RNA-Binding Proteins (RBPs)

In addition to proteins, EVs are vehicles of multiple types of cargo, such as RNAs. The secreted RNAs can be both coding and non-coding, including messenger RNAs (mRNAs) as well as small RNAs (sRNAs), and evidence of their effect on recipient cells is increasing [79]. This effect is related to the role each type of RNA fulfills in the secreting cell, i.e., vesicular mRNAs can be translated in recipient cells, while sRNAs exert gene expression regulation. The association of vesicular RNAs with proteins has been mostly studied in EVs from mammalian cell lines [80], and the role that these proteins fulfill in RNA export, stability, intracellular fate and functionality in recipient cells is a complex and exciting field, especially in the communication between pathogens and hosts. Regarding parasite EVs, they mostly carry sRNAs [81], though the presence of other RNA types has been studied in some cases [46,82,83].

**Figure 5:**
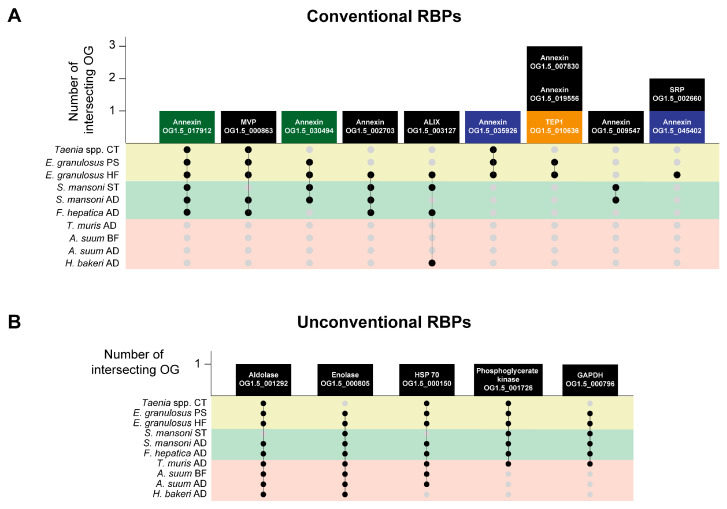
Conventional (**A**) and unconventional (**B**) RNA-binding proteins (RBPs) secreted in EVs from parasitic helminths. RBPs in black have human/mouse orthologues; while orange, green and blue proteins are specific to platyhelminth species (parasitic and free-living), parasitic platyhelminths and cestodes, respectively. Black and grey dots indicate presence or absence, respectively, in the genus/species described in the corresponding row. A connecting line indicates intersection between parasites with black dots, and the number on a bar indicates the number of intersecting OGs. Colored panels indicate the taxon: yellow for Cestoda, green for Trematoda and red for Nematoda. CT: Cysticercus, PS: Protoscolex, HF: fertile Hydatid Fluid, ST: Schistosomulae, AD: Adult, BF: Body Fluid.

From the 20 vesicular RBPs experimentally identified in vertebrate EVs [80], nine present orthologues in the three groups of parasitic helminths (Supplementary Table S9); however, only MVP (OG1.5_000863) and Alix (OG1.5_003127) were detected in parasitic platyhelminth EVs (Supplementary Table S4, Figure 5A). In addition, parasitic platyhelminth EVs carry proteins containing functional domains that are identical to some of the vertebrate vesicular RBPs but with a level of sequence divergence that prevented clustering in the same OGs. Such proteins are annexins, signal recognition particle protein (SRP) and Telomerase protein component 1 (TEP-1) (Supplementary Table S4). Interestingly, some of these proteins are only conserved among platyhelminth species (parasitic and free-living), parasitic platyhelminths or cestodes (Figure 5A), suggesting flatworms evolved distinctive RNA metabolism intermediaries that may be involved in the modulation of the environment, depending on the lifestyle of each species. 

In the EVs from nematode species, only ALIX (OG1.5_003127) was identified in the samples from *H. bakeri* adults. This result may be related to the processing pipeline used in this work that, in some cases, excluded proteins in the ID conversion step, since it has been soundly demonstrated that this species secretes a worm-specific AGO protein (WAGO) that is associated with a specific type of nematode sRNAs [84]. On the other hand, it is also possible that worms have evolved highly divergent unconventional RBPs that require biochemical characterization.

In this sense, many unconventional RBPs have been identified in recent years. These proteins are called “unconventional” because they associate with RNA but lack known RNA-binding domains (RBDs) and, in some cases, correspond to metabolic moonlighting enzymes [85]. One of the most characterized unconventional RBPs is GAPDH (OG1.5_000796) [85], whose ability to bind RNA has been studied in in vitro and in vivo systems and constitutes a commonly found protein in EVs, including those from helminth parasites (Supplementary Table S8). Of note, GAPDH controls cytokine production in T cells by binding to the AU-rich region in the 3’UTR mRNA and reducing protein translation [86]. Since helminth infections are usually associated with permissive Th2 responses, it would be interesting to assess whether T cell internalization of EVs carrying parasitic GAPDH may inhibit INF-γ production, a key Th1 polarizing cytokine [87]. Other enzymes that are commonly detected in EVs that also behave as RBPs are enolase (OG1.5_000805), aldolase (OG1.5_001292), HSP 70 (OG1.5_000150) and phosphoglycerate kinase (OG1.5_001726) [88,89].

It is worth mentioning that all these unconventional RBPs have been identified using methods focused on the detection of polyadenylated RNAs; hence, there is still limiting information regarding unconventional sRNA-binding RBPs.

### 3.6. EVs as a Source of Vaccine Antigens

In recent years, EVs secreted/excreted by helminth parasites have shown promising potential as vaccine candidates in immunoprophylactic strategies [90]. Interestingly, many of the proteins present in parasitic EVs were previously tested as subunit vaccine candidates (Supplementary Table S10), and some of them induced both homologous and heterologous immunity, i.e., against parasites other than the species that originated the antigens used for the vaccine formulation [91,92]. In this respect, proteins that successfully induced prophylactic immunity in the context of helminth infections were detected in EVs from both platyhelminths and nematodes. In mice challenged with *S. mansoni* cercariae, immunization with recombinant fructose-bisphosphate aldolase (OG1.5_001292) induced protection levels around 60% and a significant reduction in the formation of hepatic granulomas [93]. This protein is a promising pan-helminth vaccine candidate, since it is conserved among cestodes, trematodes and nematodes (Table 1); it constitutes a classic EV marker across evolution; and its presence in the EVs from the 7 species analyzed here suggests it has a predominant role in the interaction with the host. Other relevant proteins shown to induce homologous protection that are detected in parasite EVs are listed in Supplementary Table S10. Among them, enolase (OG1.5_000805) is another EV marker that has been proven to induce homologous protection against *Fasciola gigantica* [94], *H. contortus* [95] and *A. suum* [96].

Parasitic platyhelminth EVs are loaded with proteases from the Calpain family (OG1.5_000592) (Supplementary Table S5). Immunization with calpain Sm-p80 from *S. mansoni* induced homologous [97,98] as well as heterologous protection against *S. japonicum* and *S. haematobium* [92]. Remarkably, the level of protection induced by this protein was similar for the three parasite species, i.e., similar reduction in adult worm loads was detected [92]. However, a strong dependance on the adjuvant system used was observed [97,98].

As mentioned in the previous sections, parasites secrete pathogen-specific proteins in EVs, which may represent attractive vaccine candidates. However, discouraging results were reported for annexin B30 from *S. mansoni* (OG1.5_017912) [99] and galectin from *H. contortus* (OG1.5_009605) [100]. These proteins were not able to induce protection despite the fact that they are exclusively conserved among parasitic Platyhelminths (annexin B30) or nematodes (galectin) (Supplementary Table S10).

It is worth mentioning that the proteins described here correspond to a subset of successful vaccine candidates for which protein IDs could be retrieved or verified. Thus, a wider spectrum of putative vaccine candidates derived from parasitic EVs is expected. In this regard, the utility of sera from animals immunized with parasite EVs as a discovery tool seems promising [15].

## 4. Conclusions

Currently, the lack of protein markers is one of the main obstacles for the helminth EV research community [101]. To further progress in this respect, we performed a comprehensive comparative analysis of the protein content of EVs from helminth parasites and presented a detailed description of selected groups of proteins related to different processes (vesicle trafficking, RNA transport, glycolytic and key metabolic proteins), as well as vaccine candidates and parasite-specific proteins of unknown function. However, the data gathered can be further interrogated to answer other specific questions. 

Due to the orthology approach used here, defined sets of proteins were shown to be consistently detected in EV preparations from organisms at different taxa levels, providing a starting point for a selective biochemical characterization. 

## Figures and Tables

**Table 1:** Evolutionary conservation of proteins secreted in EV from helminth parasites.

Proteins Secreted in EV from	Ortholog Group	Protein Description	Orthologues																			
			Hsa/ Mmu	Sme	Cae	Egr	Emu	Tas	Tsa	Sma	Fhe	Ovi	Gsa	Asu	Bma	Dim	Hco	Hpo	Nbr	Tci	Tmu	Tsu
Cestoda + Trematoda + Nematoda	OG1.5_000110	Actin																				
	OG1.5_000150	Heat shock protein 70																				
	OG1.5_000216	Heat shock protein 90/Endoplasmin																				
	OG1.5_000805	Enolase																				
	OG1.5_001292	Fructose-bisphosphate aldolase																				
Cestoda + Trematoda	OG1.5_000551	Otoferlin/Myoferlin																				
	OG1.5_000592	Family C2 unassigned peptidase (C02 family)/Calpain																				
	OG1.5_000646	Na+/K+-transporting ATPase subunit alpha																				
	OG1.5_000731	L-lactate dehydrogenase																				
	OG1.5_000863	Major vault protein																				
	OG1.5_001170	Ectonucleotide pyrophosphatase/phosphodiesterase																				
	OG1.5_001443	Plastin 3/Fimbrin																				
	OG1.5_001898	Cathepsin B-like peptidase																				
	OG1.5_002072	Receptor Mediated Endocytosis family member																				
	OG1.5_002703	Annexin																				
	OG1.5_002906	Ras protein Rab 27A																				
	OG1.5_002908	Rab GDP dissociation inhibitor																				
	OG1.5_003543	Rab																				
	OG1.5_003615	BRO1 domain containing protein BROX																				
	OG1.5_003620	Programmed cell death protein																				
	OG1.5_004233	Nascent polypeptide associated complex subunit																				
	OG1.5_004775	Ras gtpase																				
	OG1.5_004819	Ras protein Rap 1b																				
	OG1.5_006108	Syndecan-binding protein syntenin																				
	OG1.5_006367	Charged multivesicular body protein 2a																				
	OG1.5_006593	Charged multivesicular body protein 5																				
	OG1.5_008557	Alpha tocopherol transfer protein																				
	OG1.5_010119	Hypothetical or predicted protein																				
	OG1.5_017912	Annexin																				
	OG1.5_023852	Tegumental protein																				
Cestoda	OG1.5_000444	Calcium-transporting ATPase																				
	OG1.5_000651	Long chain fatty acid coenzyme A ligase 1/5																				
	OG1.5_000774	Solute carrier family 5/Na+:glucose cotransporter 2/Na+:myo inositol cotransporter																				
	OG1.5_000781	V-type proton ATPase subunit a																				
	OG1.5_002430	Syntaxin 1a																				
	OG1.5_003065	Ras gtpase																				
	OG1.5_005812	Ras protein rab 8b																				
	OG1.5_026801	Major egg antigen p40 Putative hsp20																				
	OG1.5_030608	Endophilin B1																				
	OG1.5_035926	Annexin																				
	OG1.5_036009	Transforming protein RhoA																				
	OG1.5_036631	Expressed conserved protein																				
	OG1.5_060851	Ras protein RABF2b																				
Trematoda	OG1.5_000586	Vacuolar ATP synthase subunit b																				
	OG1.5_004511	Glutathione S transferase																				
	OG1.5_005173	Proteasome subunit alpha type																				
	OG1.5_005613	Leucyl aminopeptidase																				
	OG1.5_014186	Putative lysosome-associated membrane glycoprotein																				
	OG1.5_015286	Putative placenta-specific protein 8 protein (C15 protein) (Onzin)																				
	OG1.5_030814	Hypothetical or predicted protein																				
Nematoda	OG1.5_002305	Small heat shock protein OV25																				
	OG1.5_004286	Peptidyl prolyl cis trans isomerase CWC27/Aldo keto reductase family 1 member B4																				
	OG1.5_004557	Vitellogenin																				
	OG1.5_005795	Intestinal acid phosphatase																				
	OG1.5_008101	Titin																				
	OG1.5_021155	Apolipophorin																				

Hsa: *Homo sapiens*, Mmu: *Mus musculus*, Sme: *Schmidtea mediterranea*, Cae: *Caenorhabditis elegans*, Egr: *Echinococcus granulosus sensu stricto*, Emu: *E. multilocularis*, Tas: *Taenia asiatica*, Tsa: *Taenia saginata*, Sma: *Schistosoma mansoni*, Fhe: *Fasciola hepatica*, Ovi: *Opisthorchis viverrini*, Gsa: *Gyrodactylus salaris*, Asu: *Ascaris suum*, Bma: *Brugia malayi*, Dim: *Dirofilaria immitis*, Hco: *Haemonchus contortus*, Hpo: *Heligmosomoides polygyrus* (renamed *H. bakeri*), Nbr: *Nippostrongylus brasiliensis*, Tci: *Teladorsagia circumcincta*, Tmu: *Trichuris muris*, Tsu: *Trichuris suis*. Colored boxes indicate presence. White boxes indicate absence.

## Data Availability

The OGs generated in this work are available at https://github.com/MCucher/Ortholog_groups.

## Supplementary Materials

The following supporting information can be downloaded at: https://www.mdpi.com/article/10.3390/life13122286/s1. Supplementary Table S1: Links to the proteomes used for the construction of ortholog groups. Supplementary Table S2: Articles reporting proteomic assays of extracellular vesicles (EVs) secreted by parasitic helminths. Supplementary Table S3: Number of ortholog groups (OG) corresponding to each species/genus and sample type. Supplementary Table S4: Proteins experimentally identified in extracellular vesicles (EVs) from each species/genus and life cycle stage/sample type that were used in this work. Ortholog group (OG) numbers, IDs and protein description present in each dataset are displayed. Supplementary Table S5: Complete set of proteins experimentally identified in extracellular vesicles (EVs) from each species/genus (all stages/sample types together). Supplementary Table S6: Proteins involved in the biogenesis of extracellular vesicles. Supplementary Table S7: Proteins of unknown function experimentally detected in helminth extracellular vesicles. These proteins belong to ortholog groups (OG) exclusively composed of proteins of unknown function. Supplementary Table S8: Glycolytic and key metabolic proteins present in extracellular vesicles from parasite helminths. Supplementary Table S9: RNA-binding proteins (RBP) present in mammalian extracellular vesicles. Supplementary Table S10: Proteins secreted in helminth extracellular vesicles and assayed as subunit vaccine candidates.
